# Chromophore-loaded CRALBP restores rod visual cycle in chromophore-deficient mice

**DOI:** 10.1016/j.omta.2026.201747

**Published:** 2026-05-12

**Authors:** Robert P. van de Werken, Janet R. Sparrow, Stephen H. Tsang

**Affiliations:** 1 Jonas Children’s Vision Care (JCVC) and Barbara & Donald Jonas Stem Cell Laboratory, New York-Presbyterian Hospital, New York, NY 10032, USA; 2 Department of Biomedical Engineering, Columbia University, New York, NY 10027, USA; 3 Department of Ophthalmology, Columbia University Irving Medical Center, New York, NY 10032, USA; 4 Department of Pathology & Cell Biology, Columbia University Irving Medical Center, New York, NY 10032, USA; 5 Columbia Stem Cell Initiative, Institute of Human Nutrition, Vagelos College of Physicians and Surgeons, Columbia University Irving Medical Center, New York, NY 10032, USA

## Main text

The visual cycle depends on continuous regeneration and trafficking of 11-*cis*-retinal, and failure of that process underlies a group of inherited retinal diseases marked by night blindness, delayed dark adaptation, and progressive photoreceptor loss. In a recent issue of *Molecular Therapy Advances*, Kolesnikov et al.^1^ test whether CRALBP, a native retinaldehyde-binding protein, can be repurposed as an exogenous carrier to bypass defective chromophore production. They show that 9-*cis*-retinal-loaded wild-type CRALBP and a redox-sensitive A212C:T250C mutant restore rod function in chromophore-deficient *Rpe65*^*−/−*^ mice *ex vivo* and after intravitreal injection *in vivo* and that the engineered mutant improves photostability of bound 9-*cis*-retinal *in vitro*.^1^ The study expands chromophore replacement from small-molecule administration to protein-enabled delivery, but it also leaves open the critical translational questions of durability, retinal distribution, and whether the engineered mutant offers a meaningful *in vivo* advantage.

Visual chromophore deficiency is an attractive therapeutic target because it links several genetically distinct disorders through a shared biochemical bottleneck. Mutations in RPE65, LRAT, RLBP1, RDH5, and related genes reduce the supply of 11-*cis*-retinal to photoreceptors, limiting pigment regeneration and slowing or preventing dark adaptation.^2^^,^^3^^,^^4^ This common pathophysiology has already made the visual cycle a proving ground for intervention. Gene replacement for RPE65 disease demonstrated that restoring a missing enzyme can improve visual function, but gene therapy remains genotype specific.^5^ Small-molecule chromophore replacement with 9-*cis*-retinoids, meanwhile, has shown that functional rescue is possible even when the canonical cycle remains blocked.^6^^,^^7^^,^^8^ The challenge is delivery: free retinoids are chemically labile, systemically distributed, and potentially toxic at higher exposure, creating interest in local carriers that can protect cargo and deliver it more selectively to the retina.

CRALBP is a logical carrier because it sits at the center of retinoid trafficking. It binds 11-*cis*-retinal, 11-*cis*-retinal, and 9-*cis*-retinal with high affinity and supports chromophore handling in the retinal pigment epithelium and Muller glia.^3^^,^^4^^,^^9^ Kolesnikov et al. exploit this biology rather than replacing it. Their A212C:T250C mutant places a reversible disulfide lock across the mobile gate of the ligand-binding pocket, with the goal of shielding 9-*cis*-retinal during extracellular transit and releasing it into a reducing environment. *In vitro*, the oxidized mutant increased the photostability half-life of bound 9-*cis*-retinal 4.3-fold relative to the reduced form.^1^ This matters because carrier-mediated chromophore therapy must preserve a light-sensitive cargo long enough to reach free opsin. The idea that a native binding protein can be engineered into a tunable depot, rather than used merely as a passive chaperone, is one of the paper’s most important conceptual contributions.

The functional data are equally notable. In isolated *Rpe65*^*−/−*^retinas, both wild-type and mutant CRALBP delivered 9-*cis*-retinal efficiently enough to restore rod responses toward levels achieved with free 9-*cis*-retinal, with super-high-molecular-weight (SHMW) fractions outperforming monomeric preparations *ex vivo*.^1^ After intravitreal injection, all four formulations—wild-type monomeric, wild-type SHMW, mutant monomeric, and mutant SHMW—produced significant recovery of scotopic ERG amplitudes and sensitivity *in vivo*.^1^ Immunohistochemical staining revealed that SHMW material reached the photoreceptor layer more broadly than expected after vitreous delivery.^1^ Most striking was the dark-adaptation phenotype: after a bleach of more than 90% of pigment, treated *Rpe65*^*−/−*^ mice recovered rod function faster than wild-type control mice.^1^ That result is artificial rather than physiologic, but that is precisely what makes it interesting. The finding indicates that a pre-positioned pool of chromophore can circumvent the rate-limiting steps of the native visual cycle and create a temporary biochemical shortcut to visual pigment regeneration.

For the field, the appeal of this strategy is its mutation-agnostic logic. A CRALBP-based carrier could, in principle, be relevant across multiple disorders in which chromophore supply is impaired but photoreceptors remain salvageable. It could also complement gene therapy rather than compete with it, potentially providing rapid functional rescue while slower molecular correction takes effect. The paper is equally clear about what has not been established. The rescue observed here is short-term, spanning days rather than weeks or months.^1^ The delivered chromophore is the more stable 9-*cis*-retinal, which forms isorhodopsin rather than native rhodopsin; the latter is sufficient for rod signaling in Rpe65 deficiency, but the consequences of chronic isorhodopsin-based vision remain uncertain.^10^ The redox-sensitive mutant improved photostability *in vitro* but did not outperform wild-type CRALBP *in vivo* under the dark-adapted conditions tested.^1^ Mechanistic questions also remain: whether the injected protein is internalized or hands off cargo extracellularly, why SHMW particles distribute as they do, and how well intravitreal delivery will translate across species with thicker inner limiting membranes.^1^

Those uncertainties should not obscure the paper’s broader contribution. The study recasts CRALBP from an endogenous visual-cycle actor into a therapeutic platform: a human retinoid-binding protein that can be loaded, formulated, and engineered to shuttle chromophore where the native cycle fails. That is a meaningful addition to the inherited retinal disease toolkit, which is increasingly moving beyond one-gene/one-vector solutions toward pathway-level and combination therapy. The next experiments are now obvious and important: durability under physiological light exposure, repeat dosing, cone rescue, larger-animal delivery, and head-to-head comparison with free retinoid or sustained-release systems. If those hurdles can be cleared, protein-based chromophore delivery may become a practical detour around visual cycle failure (Table 1).

**Table 1:** What the study establishes and what must come next

Aspect	What the study shows	Why it matters
Carrier design	human CRALBP can be loaded with 9-*cis*-retinal and delivered as monomeric or SHMW formulations	uses an endogenous retinoid-binding protein as a therapeutic shuttle rather than relying only on free retinoid
Protein engineering	A212C:T250C introduces a reversible redox-sensitive lock in the mobile gate and increases *in vitro* photostability of bound 9-*cis*-retinal by ∼4.3-fold	demonstrates that cargo protection can be tuned at the protein level
Functional rescue	wild-type and mutant CRALBP restore rod responses *ex vivo* and after intravitreal injection in *Rpe65*^*−/−*^mice	supports a mutation-agnostic chromophore replacement strategy
Key unanswered questions	remaining unsolved are durability, mechanism of retinal distribution and cargo transfer, repeat dosing, cone rescue, larger-eye translation, and any *in vivo* advantage of the mutant remain unresolved	defines the translational experiments now needed

## Acknowledgments

J.C.V.C. is supported by the 10.13039/100000002National Institutes of Health through grants U01 EY030580, U01EY034590, R24EY028758, R24EY027285, 5P30EY019007, R01EY033770, R01EY018213, and R01EY024698; the 10.13039/100001116Foundation Fighting Blindness TA-GT-0321-0802-COLU-TRAP; Richard Jaffe; NYEE Foundation; the 10.13039/100025659Rosenlund Family Foundation; Schneeweiss Stem Cell Fund; the Gebroe Family Foundation; Piyada Phanaphat fund; the Research to Prevent Blindness (RPB) Physician-Scientist Award; and unrestricted funds from 10.13039/100001818RPB, New York, NY, USA.

## Declaration of interests

The authors declare no competing interests.

## References

[1] Kolesnikov A.V., Aeschimann W., Cullity J., Halabi M., Kiser P.D., Stocker A., Kefalov V.J. (2026). Chromophore-loaded CRALBP mutant proteins restore rod function in chromophore-deficient mice. Mol. Ther. Adv..

[2] Saari J.C., Bredberg L., Garwin G.G. (1982). Identification of the endogenous retinoids associated with three cellular retinoid-binding proteins from bovine retina and retinal pigment epithelium. J. Biol. Chem..

[3] Saari J.C., Nawrot M., Kennedy B.N., Garwin G.G., Hurley J.B., Huang J., Possin D.E., Crabb J.W. (2001). Visual cycle impairment in cellular retinaldehyde-binding protein knockout mice results in delayed dark adaptation. Neuron.

[4] Bassetto M., Kolesnikov A.V., Lewandowski D., Kiser J.Z., Halabi M., Einstein D.E., Choi E.H., Palczewski K., Kefalov V.J., Kiser P.D. (2024). Dominant role for pigment epithelial CRALBP in supplying visual chromophore to photoreceptors. Cell Rep..

[5] Costa B.L.D., Quinn P.M.J., Wu W.H., Liu S., Nolan N.D., Demirkol A., Tsai Y.T., Caruso S.M., Cabral T., Wang N.K., Tsang S.H. (2024). Targeting miR-181a/b in retinitis pigmentosa: implications for disease progression and therapy. Cell Biosci..

[6] Van Hooser J.P., Aleman T.S., He Y.G., Cideciyan A.V., Kuksa V., Pittler S.J., Stone E.M., Jacobson S.G., Palczewski K. (2000). Rapid restoration of visual pigment and function with oral retinoid in a mouse model of childhood blindness. Proc. Natl. Acad. Sci. USA.

[7] Koenekoop R.K., Sui R., Sallum J., van den Born L.I., Ajlan R., Khan A., den Hollander A.I., Cremers F.P.M., Mendola J.D., Bittner A.K. (2014). Oral 9-cis retinoid for childhood blindness due to Leber congenital amaurosis caused by RPE65 or LRAT mutations: an open-label phase 1b trial. Lancet.

[8] Scholl H.P.N., Moore A.T., Koenekoop R.K., Wen Y., Fishman G.A., van den Born L.I., Bittner A., Bowles K., Fletcher E.C., Collison F.T. (2015). Safety and proof-of-concept study of oral QLT091001 in retinitis pigmentosa due to inherited deficiencies of RPE65 or LRAT. PLoS One.

[9] Lima de Carvalho J.R., Kim H.J., Ueda K., Zhao J., Owji A.P., Yang T., Tsang S.H., Sparrow J.R. (2020). Effects of deficiency in the *RLBP1*-encoded visual cycle protein CRALBP on visual dysfunction in humans and mice. J. Biol. Chem..

[10] Fan J., Rohrer B., Moiseyev G., Ma J.X., Crouch R.K. (2003). Isorhodopsin rather than rhodopsin mediates rod function in RPE65 knock-out mice. Proc. Natl. Acad. Sci. USA.

